# SPP1 Regulates Radiotherapy Sensitivity of Gastric Adenocarcinoma via the Wnt/Beta-Catenin Pathway

**DOI:** 10.1155/2021/1642852

**Published:** 2021-07-27

**Authors:** Gangyi Sun, Ziyi Shang, Wenjia Liu

**Affiliations:** ^1^Department of Massage, The Affiliated Hospital of Shandong University of Traditional Chinese Medicine, Jinan 250000, China; ^2^Treating Potential Diseases Branch, The Affiliated Hospital of Shandong University of Traditional Chinese Medicine, Jinan 250000, China; ^3^Department of Oncology, The Affiliated Hospital of Shandong University of Traditional Chinese Medicine, Jinan 250000, China

## Abstract

**Purpose:**

Radiotherapy has been widely applied for the treatment of locally advanced and metastatic gastric adenocarcinoma (GAC). The aberrant expression of secreted phosphoprotein 1 (SPP1) is involved in radiosensitivity in a variety of cancers. The present study aims to characterize the clinical significance of SPP1 expression in GAC and its role and underlying mechanism of radiosensitivity.

**Methods:**

The SPP1 expression in GAC tissues and pericarcinomatous tissues was determined by QRT-PCR and immunohistochemistry, and the SPP1 expression in GAC cell lines (BGC823, AGS, and SGC7901) and normal human gastric epithelial cell line (GES-1) was determined by western blot. *T*-test, one-way ANOVA, Cox regression model, and Kaplan–Meier plotter were applied to further assess the association between SPP1 expression and the prognosis of the patients with GAC. After irradiation and transfection with si-SPP1 combined with or without Wnt/*β*-catenin pathway inhibitor (XAV939), western blot, transwell, flow cytometry, and TOP-flash reporter assay were applied to detect DNA damage, invasion, apoptosis, cell cycle, and activation of Wnt/*β*-catenin pathway, respectively.

**Results:**

SPP1 mRNA and protein levels in GAC tissues were both dramatically higher than those in pericarcinomatous tissues. SPP1 overexpression was positively associated with tumor size, nodal status, and histological grade of GAC patients. SPP1 overexpression, depth of invasion, and nodal status were independent prognostic factors for the patients. High SPP1 expression was negatively related to the overall survival in patients with GAC. We found that SPP1 knockdown enhanced the radiosensitivity of GAC cell lines (AGS and SGC7901). Increasing H2AX phosphorylation, apoptosis and G2/M phase arrest, and decreasing invasion were observed after the administration of si-SPP1 and irradiation. Radiosensitivity of SPP1 was mainly dependent on the Wnt/*β*-catenin signal pathway. XAV939 could enhance these phenomena induced by irradiation combined with SPP1 knockdown.

**Conclusion:**

This study demonstrates that SPP1 suppresses Wnt/*β*-catenin signaling to enhance the radiosensitivity of GAC via inhibiting invasion and accelerating DNA damage, G2/M phase arrest, and apoptosis.

## 1. Introduction

Gastric cancer (GC) is an aggressive malignancy with an extremely poor prognosis and a high incidence [1]. The incidence of gastric adenocarcinoma (GAC) accounts for 95 percent of gastric malignant tumors [2]. Accumulated evidence has demonstrated that radiotherapy has a role in the management of neoadjuvant, adjuvant, and palliative treatment of GC [3, 4]. However, because of the low radiosensitivity of GC, no difference in survival was observed in GAC patients receiving radiotherapy [5, 6]. Therefore, it is urgent and imperative to find an effective radiosensitizer to enhance the curative effect of radiotherapy and alleviate its toxicity to the tissues and organs around the radiation field.

Secreted phosphoprotein 1 (SPP1), which is also known as bone sialoprotein 1, osteopontin (OPN), early T-lymphocyte activation 1, and Eta-1 protein, controls the cell growth, proliferation, apoptosis, and migration [7]. High plasma concentrations of SPP1 are correlated with a poor prospect for patients with head and neck cancer after radiotherapy, which can prognosticate clinically relevant hypoxia, and might determine patients who will benefit from modifying hypoxia during radiotherapy [8]. Studies have indicated that SPP1 is elevated in many malignancies, such as ovarian cancer [9], cervical cancer [10], and breast cancer [11]. And, high SPP1 expression level at the end of radiotherapy is correlated with poor survival, such as glioma [12] and non-small-cell lung cancer [13]. However, the role of SPP1 in radiosensitivity in GAC remains unclear. Consequently, the association between SPP1 and radiosensitivity in GAC needs to be investigated further.

In this study, we found that high SPP1 was associated with poor prognosis in GAC patients. We observed that SPP1 knockdown pretreatment increased H2AX phosphorylation, apoptosis and G2/M phase arrest, and decreased invasion in response to irradiation in AGS and SGC7901 cells. Additionally, we noticed that SPP1 could activate the Wnt/*β*-catenin signal pathway in GAC cells through TOP-flash reporter assay and western blot validation, and pretreatment with Wnt/*β*-catenin signal pathway inhibitor (XAV939) sensitized GAC cells to IR.

## 2. Methods

### 2.1. Patients

A total of 198 tissue specimens, including 72 cases of adjacent tissues (3-4 cm from the tumor tissue) and 126 cases of gastric adenocarcinomas, were collected from patients with GAC who underwent surgery from September 2010 to September 2015 at the Affiliated Hospital of Shandong University of Traditional Chinese Medicine, Jinan, China. The specimens were confirmed by immunohistochemistry (IHC) and included in the clinical/prognostic analysis. Participants who met the following criteria were included: histologically confirmed diagnosis of GAC, no history of prior anticancer therapies, underwent radical surgery, and complete follow-up data available. Written informed consents were signed by all participants. Ethical approval to conduct this study was obtained from the ethics committee of the Affiliated Hospital of Shandong University of Traditional Chinese Medicine.

### 2.2. Immunohistochemistry

All specimens were routinely fixed in 10% buffered formalin and embedded in paraffin. And, the 4-*μ*m-thick tissue sections that were cut from wax blocks were prepared on APES-coated glass slides. Slides were deparaffinized in xylene, rehydrated in graded ethanol, and immersed in 3% hydrogen peroxidase-methanol for 15 minutes to eliminate endogenous peroxidase activity. A microwave antigen retrieval procedure was performed for 5 min in 10 mM citrate buffer (pH 6.0). The sections were incubated with a primary anti-SPP1 antibody (1 : 200, ab8448, Abcam, Shanghai, China) at 4°C for overnight. After incubation with HRP-conjugated secondary antibody (1 : 50, ab6721, Abcam) at room temperature for 30 minutes, they are visualized by using 3,3′-diaminobenzidine and then counterstained with hematoxylin for 3 min.

Two experienced pathologists blinded to the clinical data were responsible for reviewing the immunoreactivity of SPP1. The score of staining intensity as well as the proportion of immunostaining positive cells was between 0–3 and 0–4, respectively. The proportions were as follows: 0, negative; 1, ≤10% positive cells; 2, >10% but ≤50% positive cells; 3, >50% but ≤75% positive cells; and 4, >75% positive cells. The staining intensity was as follows: 0, absent; 1, weak; 2, moderate; and 3, strong. The two scores were multiplied to obtain an overall protein expression score. For statistical analysis, the final evaluation criteria of SPP1 expression were as follows: 0–4 as low expression and 5–12 as high expression.

### 2.3. RNA Extraction and RT-qPCR

Total RNA from GAC tissues and pericarcinomatous tissue was extracted by TRIzol solution (Invitrogen, CA, USA). To synthesized cDNA, PrimeScript™ IV 1st strand cDNA Synthesis Mix (Takara, Dalian, China) was applied. The QPCR was carried out by the SYBR Premix ExTaq TM II (Takara, Dalian, China) by ABI 7900 qRT-PCR system (Applied Biosystems, Foster City, CA, United States). The cDNA was then subjected to qRT-qPCR by SYBR Premix ExTaq TM II (Takara, Dalian, China) to evaluate the relative mRNA levels of SPP1 on an Applied Biosystems real-time PCR machine (ABI 7900HT, CA, USA), and *β*-actin was applied as the internal control. And, the relative mRNA level was calculated by utilizing the 2^−ΔΔCt^ method.

Primer sequence:

  SPP1 F: 5′-TTTGTTGTAAAGCTGCTTTTCCTC-3′
  R: 5′-GAATTGCAGTGATTTGCTTTTGC-3′
 
*β*-Actin F: 5′-CTCTCTCTACCTACATCTCTACTAAAA-3′
  R: 5′-AACTCTAACTCTCTCTCTAACTACTTCTC-3′

### 2.4. Cell Culture

Normal human gastric epithelial cell line (GES-1) and human stomach gastric adenocarcinoma cell line (AGS, SGC7901, and BGC823) were obtained from the Center for Chinese Typical Cultures Preservation, Wuhan University. All cell lines were cultured in 1640 media supplemented with 10% fetal bovine serum (FBS) at 37°C in 5% CO_2_ incubator. In the subsequent experiments, cells were pretreated with or without a *β*-catenin inhibitor XAV939 (10 *µ*M, Sigma-Aldrich, Darmstadt, Germany) for 48 h and then were exposed to 4 Gy for 24 h with X-ray irradiator RS2000 (RAD SOURCE, USA).

### 2.5. Transfection

The AGS and SGC7901 cells (5 × 10^6^/ml) were cultivated at 37°C under 5% CO_2_ water-saturated atmosphere in 1640 medium supplemented with 10% FBS. Two si-RNAs were synthesized by GenePharma (Shanghai, China) and transfected into GAC cell lines using lipofectamine 3000 reagent (siRNA: lipofectamine = 10 nM:1 *µ*L) (Invitrogen, Shanghai, China) for 24 h. Then, serum-free transfection solution was discarded and replaced with 1640 medium supplemented with 10% FBS for further culture. After 48 h, cellular proteins can be extracted to verify the knockdown efficiency.

The siRNA sequences were as follows:

  Si-SPP1-1, 5′-CGAUCGAUAGUGCCGAGAAGC-3′
  Si-SPP1-2, 5′-AGCUAGUCCUAGACCCUAAGA-3′

### 2.6. Western Blot

Total protein was isolated by RIPA lysis buffer (Abcam, Shanghai, China), and then the protein concentration was measured using a Pierce BCA assay (Abcam, Shanghai, China). Protein (40 *μ*g) was separated on SDS-PAGE and transferred to polyvinylidene (PVDE) membrane. After being blocked with 5% nonfat milk at room temperature for 1 h, the membranes were incubated with the primary antibodies anti-SPP1 (1 : 1000, ab8448), *γ*-H2AX (1 : 1000, ab81299), Bax (1 : 1000, ab32503), Bcl-2 (1 : 1000, ab32124), c-JUN (1 : 1000, ab40766), c-myc (1 : 1000, ab32072), cyclin-D1 (1 : 1000, ab16663), *β*-catenin (1 : 1000, ab223075), and *β*-actin (ab8227, 1 : 2000) overnight and then probed with HRP-conjugated anti-IgG antibody (1 : 1000, ab133470, all obtained from Abcam, Shanghai, China) for 1 h. Immunoreactive bands were visualized with an enhanced chemiluminescence system (Thermo Scientific, Shanghai, China).

### 2.7. Transwell Assay

Total 1 × 10^4^ cells (AGS and SGC7901) were seeded into Matrigel upper chambers (BD Bioscience, USA). The lower chamber contained a complete medium. After 24 hours of culture, the cells passing through the Matrigel membrane were fixed in methanol, stained, and counted in five fields under a microscope (Olympus, Japan).

### 2.8. Cell Cycle Detection

A total of 2 × 10^5^ cells/well were plated into a six-well plate. Before any treatment, the cell culture medium was changed to a serum-free medium to culture the cells for 12 h, ensuring it enters a similar phase. IR with or without siRNA transfection and XAV939 treatment was carried out as described in the previous methods. After 48 h, the cells were centrifuged, collected, then were fixed in 700 *µ*l of 75% ethanol (−20°C precooled). Subsequently, the cells were suspended in 400 *µ*l of propidium iodide (PI) solutions (50 *µ*g/ml) containing 100 *µ*l of 1 mg/ml RNAse (Solarbio, Shanghai, China) for 10 min. Cellular DNA content was analyzed using flow cytometer (Accuri C6, BD Biosciences).

### 2.9. Cell Apoptosis Assay

A total of 2 × 10^5^ cells/well were plated in 6-well plates and incubated for 24 h. GAC cells were transfected with siRNA combined with XAV939, followed by IR (4 Gy). Subsequently, all cells were collected and stained with 100 ul of 1× binding buffer, 5 *µ*l of Annexin V-FITC, and 5 ul of Annexin PI in the dark for 15 min at 4°C. Apoptotic cells were analyzed using a flow cytometer (BD Biosciences).

### 2.10. Luciferase Reporter Assay

A total of 1 × 10^5^ cells/well were plated in a 24-well plate and incubated with RPMI-1640 medium at 37°C for 24 h. Cells were transfected with a 0.8 *µ*g TOP-flash or FOP-flash vector and 0.02 *μ*g Renilla luciferase vector as an internal control (EMD Millipore, Billerica, MA, USA). After transfection for 24 h, cells were harvested in passive lysis buffer (Promega), and the reporter activities were assayed by Dual-Luciferase Assay System kit (Promega Corporation). Renilla reniformis luciferase expression was used for normalization.

### 2.11. Statistical Analysis

The significance among two or more comparisons was analyzed by a two-tailed Student's *t*-test or one-way ANOVA. All data were expressed as mean ± standard deviation (SD) and a *p* value <0.05 was indicated statistically significant.

## 3. Results

### 3.1. SPP1 Is Upregulated in Gastric Adenocarcinoma

**Figure 1:**
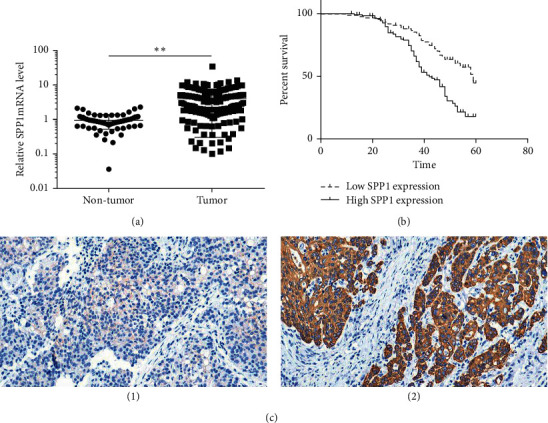
SPP1 was elevated in GAC tissues. (a) The SPP1 mRNA levels in GAC tissues and adjacent normal tissues were measured by RT-qPCR vs. normal tissues, ^*∗∗*^*p* < 0.01. (b) The SPP1 protein levels in GAC tissues and adjacent normal tissues were measured by immumohistochemical staining. (c) (1) low expression; (2) high expression.

RT-qPCR was carried out to measure SPP1 mRNA levels in GAC tissues and adjacent normal tissues. SPP1 was overexpressed in GAC tissues compared with normal gastric tissues (*p* < 0.01, Figure 1(a)). This result was confirmed by detecting the level of SPP1 protein. As shown in Figure 1(b), negative staining could be discovered in adjacent normal tissues. It was found that SPP1 protein was mainly located at the cytoplasm of the GAC cell. 169 GAC tissues (85.4%) expressed SPP1. Among the 198 GAC tissues, 110 samples (55.6%) expressed high levels, and 88 samples (44.4%) expressed low levels of SPP1.

### 3.2. Correlations between SPP1 Expression and Clinicopathological Characteristics

To implore the clinical value of SPP1 in GAC, we further examined the correlation between SPP1 expression and the clinicopathological parameters, including age, gender, tumor location, lymphatic/venous invasion, depth of invasion, tumor size, histological grade, and nodal status of patients with GAC, as shown in Table 1. No significant correlations were observed between SPP1 expression and age, gender, tumor location, lymphatic/venous invasion, and depth of invasion (*p* > 0.05) of GAC patients. Interestingly, SPP1 overexpression was positively associated with tumor size (*p*=0.035), histological grade (*p*=0.003), and nodal status (*p*=0.002) of GAC patients.

Univariate analysis revealed the following: SPP1 overexpression (hazard ratio (HR), 2.399; *p* < 0.001), histological grade (HR, 1.782; *p*=0.005), lymphatic/venous invasion (HR, 1.605; *p*=0.022), depth of invasion (HR, 1.471; *p*=0.002), and nodal status (HR, 1.672; *p* < 0.001) (Table 2). Further multivariate analysis presented that SPP1 overexpression (HR, 3.303; *p*=0.001), depth of invasion (HR, 0.511; *p*=0.025), and nodal status (HR, 1.432; *p*=0.001) were independent prognostic factors for the patients (Table 2). Survival analysis showed that the overall survival (OS) of all GAC patients with SPP1 overexpression was dramatically less than for patients with SPP1 low expression (Figure 1(c)).

### 3.3. Suppression of SPP1 Expression Affected DNA Damage, Invasion, and Apoptosis of GAC Cells

**Figure 2:**
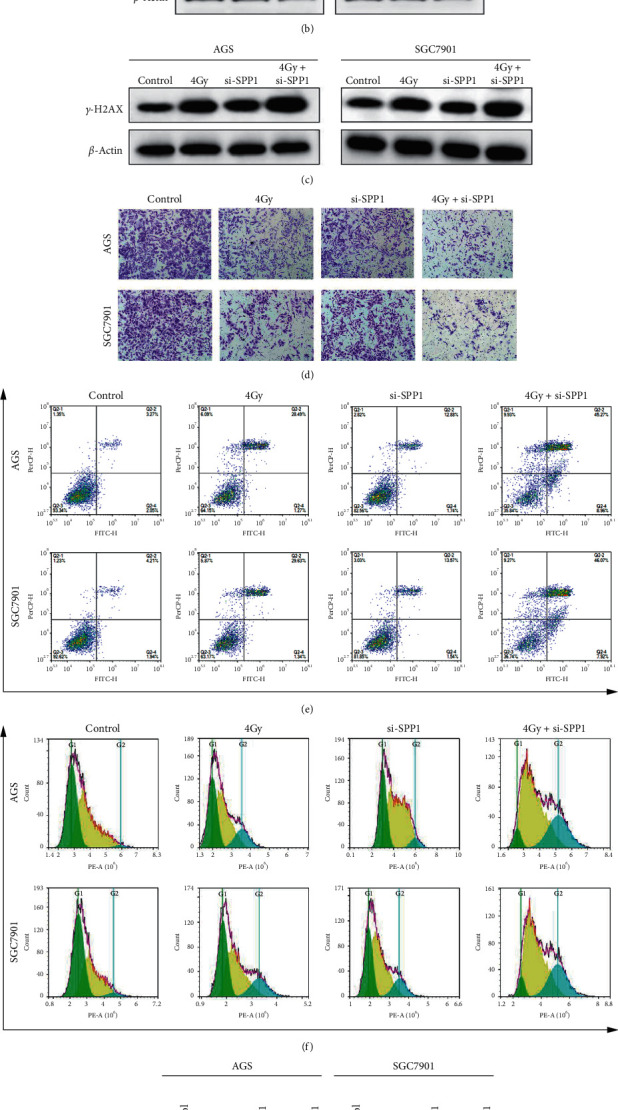
Suppression of SPP1 expression affected DNA damage, invasion, and apoptosis of GAC cells. (a) The western blot results verified that SPP1 was overexpressed in AGS and SGC7901 cells compared with control GES-1 cells. (b) The transfected efficiency of si-SPP1 in GAC cells. (c) The phosphorylation of H2AX in AGS and SGC-7901 cells was measured by western blot. (d) The invasiveness in AGS and SGC-7901 cells was measured by transwell assay. (e) The apoptosis ratio in AGS and SGC-7901 cells was measured by flow cytometry. (f) The cell cycle in AGS and SGC-7901 cells was measured by flow cytometry. (g) The apoptosis-related protein in AGS and SGC-7901 cells was measured by western blot.

The western blot results verified that SPP1 was overexpressed in AGS and SGC7901 cells compared with control GES-1 cells (Figure 2(a)). To evaluate the effect of SSP1 on radiosensitivity in GAC, we transfected the GAC cell lines AGS and SGC-7901 with SPP1-specific siRNAs or a control siRNA (si-NC). Two siRNAs targeting SPP1 were tested (si-SPP1#1 and si-SPP1#2). Si-SPP1#1 was the most effective in suppressing SPP1 expression and was adopted for subsequent study (Figure 2(b)). To investigate the effect of SPP1 on radiotherapy-mediated invasion and apoptosis, we performed experiments in vitro. The phosphorylation of H2AX was elevated in AGS and SGC-7901 cells after irradiation compared with that in the control cells, whereas in the si-SPP1 group, it was enhanced upon irradiation, indicating that SPP1 may exert an important function in DNA damage repair via inducing the phosphorylation of SPP1 (Figure 2(c)). Additionally, the invasive capacity of AGS and SGC-7901 cells was prominently restricted compared with that in the control cells. Concurrently, SPP1 depletion enormously reinforced the effect of irradiation-induced reduction in invasiveness (Figure 2(d)). Compared with the control cells (5.68% ± 0.36, 6.47% ± 0.44), AGS and SGC-7901 cells exposed irradiation and led to markedly increased apoptosis ratio (15.59 ± 0.50%, 16.08 ± 0.87%). Concurrently, transfection of AGS and SGC-7901 cells with si-SPP1 enhanced the augment in apoptosis ratio induced by irradiation (55.12 ± 0.87%, 54.46 ± 0.59%) (Figure 2(e)). There is a perceptible enrichment of GAC cells observed in the G2/M phase when AGS and SGC-7901 cells exposed irradiation, and the suppression of SPP1 remarkably enhanced the irradiation-induced G2/M phase arrest (Figure 2(f)). The irradiation notably increased Bax expression and decreased Bcl-2 expression, while knockdown of SPP1 significantly accelerated the irradiation-induced increase in Bax expression and decrease in Bcl-2 expression (Figure 2(g)). The observations indicated that suppression of SPP1 tremendously enhanced the radiosensitivity of GAC cell lines via inhibiting invasion and accelerating DNA damage, G2/M phase arrest, and apoptosis.

### 3.4. SPP1 Enhanced the Radiosensitivity of GAC Cell Lines via Wnt/*β*-Catenin Pathway

**Figure 3:**
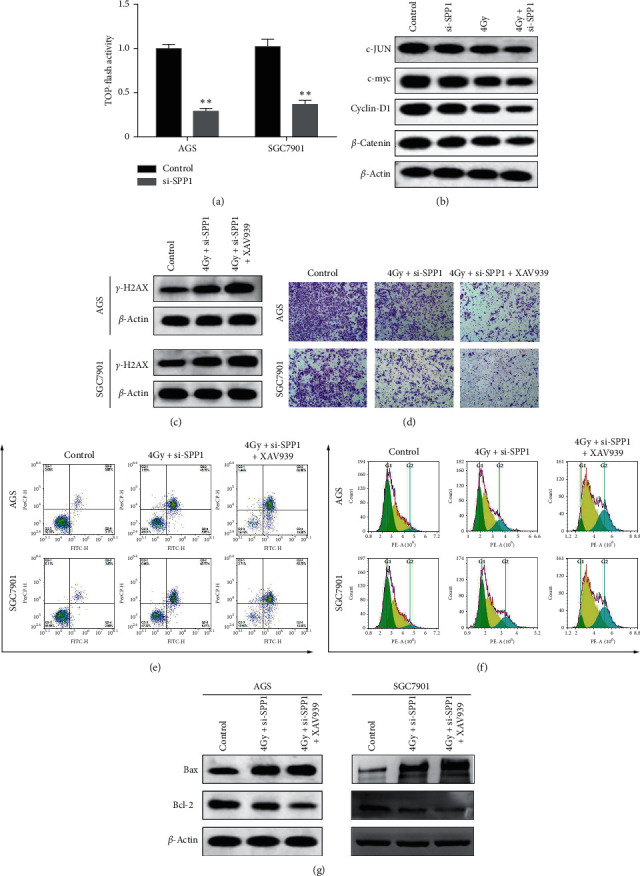
SPP1 enhanced the radiosensitivity of GAC cell lines via the Wnt/*β*-catenin pathway. (a) The Wnt pathway in AGS and SGC-7901 cells was measured by TOP-flash/FOP-flash reporter system vs. control group, ^*∗∗*^*p* < 0.01. (b) The Wnt/*β*-catenin pathway-related protein in AGS and SGC-7901 cells was measured by western blot. (c) The phosphorylation of H2AX in AGS and SGC-7901 cells was measured by western blot. (d) The invasiveness in AGS and SGC-7901 cells was measured by transwell assay. (e) The apoptosis ratio in AGS and SGC-7901 cells was measured by flow cytometry. (f) The cell cycle in AGS and SGC-7901 cells was measured by flow cytometry. (g) The apoptosis-related protein in AGS and SGC-7901 cells was measured by western blot.

Next, we examined the effect of SPP1 knockdown on the activity of the Wnt pathway enabling the TOP-flash/FOP-flash reporter system. SPP1 knockdown induced a 40% to 50% decrease in Wnt-activity in AGS and SGC-7901 cells (Figure 3(a)). The irradiation downregulated the expression of endogenous *β*-catenin, c-JUN, c-myc, and cyclin-D1 both in AGS and SGC-7901 cells, and SPP1 knockdown enormously enhanced the irradiation-induced suppression effect on the Wnt/*β*-catenin pathway (Figure 3(b)). To further verify the role of the Wnt/*β*-catenin pathway in radiosensitivity of GAC, we pretreated cells with the recombinant Wnt inhibitors XAV939. In western blot analysis, inhibitors XAV939 resulted in the reinforcement in the phosphorylation of H2AX-induced by irradiation combined with SPP1 depletion (Figure 3(c)). Additionally, XAV939 enormously reinforced the effect of irradiation combined with SPP1 depletion-induced reduction in invasiveness (Figure 3(d)). Treatment with XAV939 (84.56% ± 1.85, 83.65% ± 0.98) in AGS and SGC7901 cell lines enhanced the augment in apoptosis ratio induced by irradiation combined with SPP1 depletion (54.30% ± 0.66, 51.91% ± 1.52) (Figure 3(e)). XAV939 remarkably enhanced the irradiation combined with SPP1 depletion-induced G2/M phase arrest (Figure 3(f)). XAV939 significantly accelerated the irradiation combined with SPP1 depletion-induced increase in Bax expression and decrease in Bcl-2 expression (Figure 3(g)). This evidence indicated that SPP1 enhanced the radiosensitivity of GAC cell lines via the Wnt/*β*-catenin pathway.

## 4. Discussion

Radiotherapy has been widely applied for the treatment of locally advanced and metastatic GAC [14, 15]. The search for an effective gene target to enhance the sensitivity of radiotherapy is a vital and urgent issue for the therapy of GAC. Previous studies revealed that SPP1 is involved in the radiosensitivity of a variety of cancers [16, 17]. In the current study, we found that SPPI overexpression was associated with poor prognosis in GAC patients, and si-SPP1 pretreatment decreased invasion and increased irradiation-induced DNA damage, cell cycle arrest, and cell death in GAC cells; the underlying mechanism involves suppression of the Wnt/*β*-catenin pathway.

It is reported that SPP1 overexpression is associated with high invasive and metastatic potential, poor prognosis, and recurrent disease for cancer patients, such as breast cancer [18] and gastric cancer [19]. In this study, we confirmed that SPP1 was elevated in GAC tissues and cell lines, and SPP1 overexpression was positively associated with tumor size, nodal status, and histological grade of GAC patients. SPP1 overexpression, nodal status, and depth of invasion were independent prognostic factors for the patients. High SPP1 expression was negatively correlated with survival time in patients with GAC. These pieces of evidence were consistent with the previous study.

In the human response to nascent DNA damage, one of the earliest events is the phosphorylation of histone H2A variant H2AX to form *γ*-H2AX [20]. Within 10–30 minutes after DNA damage induction [21], the reaction is highly amplified, and many H2AX molecules flanking the DNA double-strand break site are phosphorylated. In the past decade, *γ*-H2AX has developed into a vital biomarker for quantifying DNA double-strand break levels in cells and tissues [22, 23]. To further investigate the role of SPP1 in radiosensitivity in GAC, we transfected GAC cell lines with SPP1 knockdown plasmid and found that knockdown of SPP1 enhanced the radiation-induced increase in DNA damage, apoptosis, and G2/M phase arrest and decrease in the invasion, which indicates that SPP1 knockdown can enhance the radiosensitivity in GAC.

Signaling transduction after irradiation plays a vital role in mediating damage repair, thereby controlling the fate of cancer cells receiving irradiation [24, 25]. Studies have found that activation of the *β*-catenin pathway can effectively accelerate the repair of DNA double-strand breaks in osteoblasts after irradiation [26], and *β*-catenin overexpression aggrandized the capability of DNA double-strand break repair in cancer cells [27]. Therefore, it is adjudged that the function of *β*-catenin in DNA damage repair may be a factor that affects cell radiosensitivity. Finding gene targets that interfere with radiation-related signaling pathways is an absorbing aim. In the current study, we found that SPP1 knockdown induced a decrease in the Wnt/*β*-catenin-activity in AGS and SGC-7901 cells through the TOP-flash/FOP-flash reporter system and western blot, indicating that the radiosensitivity of SPP1 was mainly dependent on the Wnt/*β*-catenin signal pathway. To further verify this hypothesis, we introduced Wnt/*β*-catenin signal pathway inhibitors (XAV939), which could enhance these phenomena induced by irradiation combined with SPP1 knockdown. This evidence revealed that SPP1 suppresses Wnt/*β*-catenin signaling to enhance the radiosensitivity of GAC via inhibiting invasion and accelerating DNA damage, G2/M phase arrest, and apoptosis.

This study is the first to indicate that SPP1 can be utilized as a gene target for enhancing radiotherapy sensitivity of gastric adenocarcinoma, triggering the Wnt/*β*-catenin pathway. However, these inclusions were only conducted at the cellular level, and animal experiments are still needed for further verification.

In conclusion, in the current study, we illustrated for the first time that the knockdown of SPP1 combined with irradiation exerted additive effects on inducing DNA damage, apoptosis and G2/M phase arrest, and suppressing invasion via the Wnt/*β*-catenin signal pathway. This indicates that SPP1 is a potential target for enhancing the efficacy of radiotherapy.

## Figures and Tables

**Table 1:** Association between SPP1 expression and clinicopathologic characteristics in patients with GAC.

Characteristics	SPP1 expression		*p* value
	Low expression (*n* = 88)	High expression (*n* = 110)	
*Gender*			0.901
Male	42	52	
Female	46	58	

*Age (years)*			0.693
<60	43	52	
≥60	45	58	

*Tumor location*			0.192
Proximal	49	51	
Distant	29	37	
Total	10	22	

*Tumor size (cm)*			0.035
<5	49	44	
≥5	39	66	

*Histological grade*			0.003
Well/moderately differentiated	51	45	
Poorly differentiated	37	65	

*Lymphatic/venous invasion*			0.845
No	58	48	
Yes	30	62	

*Depth of invasion*			0.894
T2	49	17	
T3	29	39	
T4a	10	54	

*Nodal status*			0.002
N1	42	10	
N2	24	41	
N3	22	59	

**Table 2:** Univariate and multivariable analysis of prognostic factors for 5-year survival in GAC.

Variables	Univariate analysis		Multivariate analysis	
	HR (95% CI)	*p* value	HR (95% CI)	*p* value
Gender	1.345 (0.899–2.012)	0.150		
Age	1.335 (0.894–1.995)	0.158		
Tumor location	1.258 (0.972–1.628)	0.081		
Tumor size (cm)	1.216 (0.889–1.662)	0.221		
Histological grade	1.782 (1.195–2.657)	0.005	1.051 (0.288–3.842)	0.940
Lymphatic/venous invasion	1.605 (1.072–2.404)	0.022	0.616 (0.183–2.076)	0.434
Depth of invasion	1.471 (1.151–1.878)	0.002	0.511 (0.283–0.921)	0.025
Nodal status	1.672 (1.288–2.171)	<0.001	1.432 (0.820–2.502)	0.001
SPP1 expression	2.399 (1.560–3.687)	<0.001	3.303 (1.673–6.523)	0.001

## Data Availability

The data used to support the findings of this study are available from the corresponding author upon request.
